# Some Notes on Medical Jurisprudence

**Published:** 1884-10-20

**Authors:** James A. Riley

**Affiliations:** New York


### SOME NOTES ON MEDICAL JURISPRUDENCE.

By James A. Riley, Esq., New York.

The question of expert evidence is a vexed one, and much has been, and much can be. said both in favor of and against the methods now in use in the courts for securing testimony of this character. A good many of the thinkers on this subject are of the opinion that the experts should be called by the court for its own information and that of the jury, instead of by one side to bolster up its own case, in which event bias is always possible, and generally probable. A curious instance of the extent to which the testimony of physicians is sought in some cases comes to us from a Michigan court, and the great interest of the matter consists in the attempt to get medical evidence, not as to the effects of wounds or disease, but as to the results upon the health of words spoken. The case was that of a person threatened with expulsion from a railroad train for using part of an excursion ticket, the other part being detached. The passenger claimed that the words and threats of the conductor affected his health, and called a physician on the trial who testified to that effect. This was regarded as an error when the case came upon appeal, and the court commented as follows upon the proposition to allow medical testimony as to the effect of mere words upon the health: "The medical evidence which was given in the case respecting the alleged assault upon the plaintiff's health seems to call for some comment. As the assault was a battery only in a technical sense, and there was no pretence of injury except such as might come from mere words—from the mere expression on the part of the conductor of a determination to put the plaintiff off the car unless he paid his fare—the proposition that it was proper to call expert witnesses to show the possibility of injurious consequences from such words to the plaintiff's health is suggestive of possibilities in the trial of cases which the trial judge may well contemplate with some solicitude. If expert evidence of the sort were admissible in this case, it is difficult to conceive of a case of assault and battery or of any other case in which vexing or provoking words are made use of where the expert witness may not become an important factor in determining the result." The decision of the court did not hold directly that the testimony as to the effect of words spoken upon the health was improper, though this was the inference; but it was decided that, if such testimony were to be admitted, other testimony, showing the passenger's weak condition of body, should be taken as proof that the words of the conductor were not entirely the cause of his sickness.

A recent case in New Hampshire brought up the question of the power of a health board to employ nurses for the proper care of persons afflicted with contagious diseases who were committed by the authorities to a pest-house, and to charge the town for such services. The court held that they had the authority to bind the town, and gave an interesting opinion, as follows: "Having power to compel the confinement of infective persons and prevent their communication with others, and having exercised that power, it became their duty to provide for the wants of those confined there. They could not shut them out from communication with their friends and with the world and deprive them of any means of obtaining assistance, and at the same time lawfully withhold necessary support and care. They could not establish a hospital without ordinary hospital accommodations, and they could not make the hospital a place of involuntary confinement and escape the duty of supplying the reasonable wants of those who, by confinement and seclusion, were prevented from applying to any one else for relief. They could not furnish relief against the will of those confined, and afterward recover the expense of them or of those liable in law to support them. Farmington *v.* Jones, 36 N. H., 271. If the health officers were obliged by law to confine persons infected with dangerous diseases, and it was their duty to nurse and support them, and they could not recover the reasonable expense from them or from their guardians, they would be remediless unless they could charge the expense upon the city. The law does not require the performance of a duty and at the same time withhold the means reasonably necessary for its performance."

The power to abate nuisances is frequently exercised by courts at the instance of health boards, and the latter are particularly active now in these times, when there is danger of cholera in every sea-board city and town. A recent instance of such activity was shown in the application made in an English case to oblige a railroad company to remove its cattle yards from a place where they were alleged to be injurious to health, and this the court directed to be done.

In a case not long since before one of the English courts, a person sued a surgeon for negligence in allowing him to suck a tube used in the operation of tracheotomy on his child for diphtheria, without warning him of the danger, with the result that the father contracted diphtheria. The result of the suit was, however, in favor of the surgeon.

A curious problem is now before an English court, viz, to decide whether a man can give away his head after it has become what one of the papers styles a dead-head. It seems that a person who had no sentimental notions about the disposal of his body after death sold his head to the village phy sician, who was to defray the expenses of the funeral of the rest of his body. The owner of the head is now dead, and the physician claims that the contract shall be carried out by the relatives of the deceased. As there is a large estate, the family are desirous of avoiding the contract, and the court has to decide which has the better claim.—*Med. Times.*

